# Nanoscale Wire Bonding of Individual Ag Nanowires on Au Substrate at Room Temperature

**DOI:** 10.1007/s40820-017-0126-8

**Published:** 2017-02-08

**Authors:** Peng Peng, Wei Guo, Ying Zhu, Lei Liu, Guisheng Zou, Y. Norman Zhou

**Affiliations:** 1 School of Mechanical Engineering and Automation, Beihang University, Beijing, 100191 People’s Republic of China; 2 International Research Institute for Multidisciplinary Science, Beihang University, Beijing, 100191 People’s Republic of China; 3 Department of Mechanical Engineering, Tsinghua University, Beijing, 100084 People’s Republic of China; 4 Centre for Advanced Materials Joining, University of Waterloo, Waterloo, ON N2L 3G1 Canada

**Keywords:** Nanojoining, Bonding, Diffusion, Interface, Nanoindentation

## Abstract

**Abstract:**

The controllable wire bonding of individual Ag nanowires onto a Au electrode was achieved at room temperature. The plastic deformation induced by pressure using nanoindentation could break the protective organic shell on the surface of the Ag nanowires and cause atomic contact to promote the diffusion and nanojoining at the Ag and Au interface. Severe slip bands were observed in the Ag nanowires after the deformation. A metallic bond was formed at the interface, with the Ag diffusing into the Au more than the Au diffused into the Ag. This nanoscale wire bonding might present opportunities for nanoscale packaging and nanodevice design.

**Graphical Abstract:**

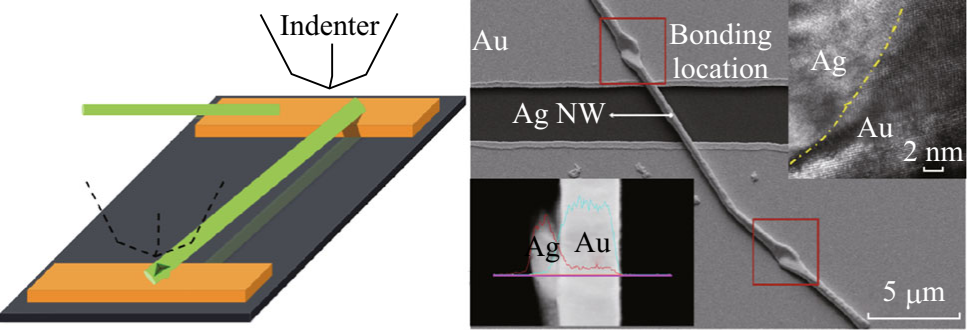

## Highlights

- A nanoscale wire-bonding of Ag nanowire with Au substrate has been realized successfully at room temperature.
- Large plastic deformation could promote the interdiffusion and nanojoining between Ag nanowire and Au electrode.
- The bonding interface has been examined and the bonding mechanism has been proposed.

## Introduction

Gold (Au) wire has been used for decades in wire bonding, a technique to interconnect an integrated circuit chip with metal leads in the semiconductor industry [1]. The cost of Au wire has significantly increased in recent years [2]. This has prompted the study and use of alternatives such as silver (Ag) [3, 4], copper (Cu) [5–7], and Ag/Au alloys [8, 9]. Cu wire suffers from oxidation issues, as well as a high hardness and Young’s modulus. Thus, it is difficult to bond. Various intermetallic compounds have been prepared that would affect the efficiency of a device and thus reduce its lifetime [10]. Currently, cost concerns are leading to wire diameter decreases, which is made possible to increase the packing density using finer pitches. Controllable bonding or welding at a submicrometer scale or nanoscale is still a great challenge [11]. Many efforts have been made to push the size limitation down to the nanoscale [12], including nanoscale resistance spot welding [13, 14], nanoscale soldering [15, 16], and ultrasonic bonding [17]. Because of the small energy requirement [11] and reactivity of nanomaterials, some new bonding methods have been reported based on novel concepts, including the cold welding of Au and Ag nanowires (NWs) by oriented attachment [18, 19], plasmonic welding of Ag NWs with plasmonic effects [20, 21], nanowelding using a scanning probe microscope [22], and optically controlled nanosoldering [23].

The thermo-compression bonding method is used for wafer bonding with diffusion [24]. Atomic contact can be achieved by simultaneously applying pressure and heat. This is usually used for large bonds [25, 26] because the pressure and heat are difficult to control at the nanoscale. Recently, the pressure bonding of individual Ag NWs with large plastic deformation under a cold condition has been demonstrated [27]. Here, we report the controllable bonding of individual Ag NWs onto Au pads using pressure at room temperature. The Ag NWs were placed into contact with the Au substrate under pressure and formed metallic bonds. This new nanoscale wire bonding method might create opportunities to direct designing nanodevices or achieve nanoscale packaging for electronics.

## Experimental

A sputtered Au electrode on a Si substrate was cleaned and used for bonding the Ag NWs onto a Au substrate. Ag NWs with a pentagonal cross section were synthesized using a previously described method [19]. A polyvinylpyrrolidone (PVP) organic layer with a thickness of 2–3 nm was fabricated on the surface of Ag NWs. Their diameter was approximately 100–300 nm. A nanoindenter (Hysitron TriboIndenter) with a Berkovich tip (diamond) and visualization system were employed for both the locating and bonding processes. The bonding forces had a range of 200–500 µN, with a loading speed of 20 µN s^−1^. The morphologies and microstructures were characterized using scanning electron microscopy (SEM, LEO 1530, Zeiss). A focused ion beam (FIB, NVision 40, Zeiss) was used to slice the Ag–Au bonding interface, which was also observed using a high-resolution transmission electron microscope (HRTEM, JEOL 2010) equipped with an energy-dispersive X-ray spectroscope (EDS).

## Results and Discussion

**Fig. 1:**
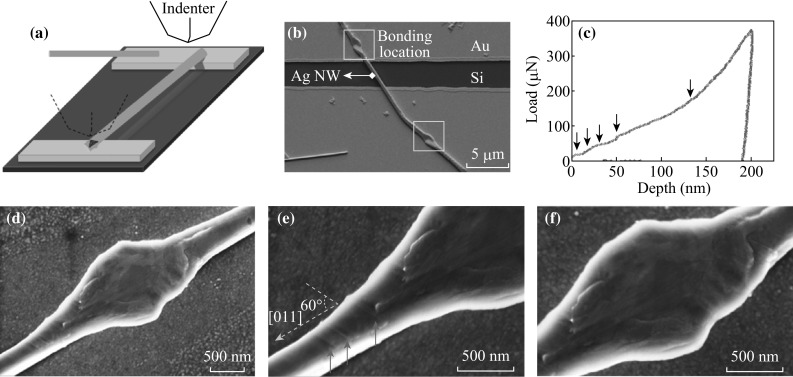
**a** Schematic illustration of nanoscale wire bonding using pressure. **b** Wire bonding of Ag NWs onto Au electrodes with two welds. **c** A typical *F*-*D* curve during bonding of Ag NWs on Au. **d** SEM image of bonding location, with high-magnification images of **e** and **f** close to the indentation area with a force of 400 µN

Figure 1a shows the scheme for the nanoscale wire-bonding process. The Ag NWs across two Au electrodes could be seen using an optical imaging lens, although the resolution was fairly low. The scanning mode of the indenter could also visualize the Ag NWs sitting on the two electrodes and assist with selecting and confirming the bonding locations. The indenter tip was placed on top of the Ag NWs and aligned at their center. A force was then loaded at a rate of 20 µN s^−1^. After reaching the maximum load, the load was unloaded at the same rate with or without a holding time. When one indentation was completed, the tip was moved to another location where the bonding was needed. This process was then repeated to complete the wire bonding at two or more bonding locations. An SEM image of the bonded wire is shown in Fig. 1b. The Ag NWs had a thickness of approximately 350 nm, and that of the Au electrode was approximately 2 µm. Figure 1c shows a typical load–displacement curve. The elastic region during loading is quite narrow (~5 nm), followed by the first “pop-in” event because of the dislocation nucleation (as indicated by the first arrow on the left). It has been reported that the stress initiation at this point is quite high (reaching the theoretical shear stress for the nucleation of dislocations [28]). The following “pop-in” marks (as shown by the second to fifth arrows) correspond to the different slip systems or bands.

During the bonding process, the depth was used as a reference to predict the bonding quality if all the alignments were good. Here, we used the rule of 1/2 the thickness of the Ag NWs to select the force. It is worth noting that if the thickness of the Ag NWs was known, the bonding process could be controlled using the displacement-control mode. If Ag NWs have a 50% plastic strain in the thickness direction, we would be confident saying that a bond can be obtained after such a large plastic deformation under pressure. The statistical results showed that a 50% plastic strain was good for bonding, whereas a plastic strain of less than 30% only resulted in an indentation and one larger than 70% would damage the NWs. Figure 1d shows the bonding location with a force of 400 µN. After bonding, the Ag NWs became twice as wide at the indent compared with the original thickness. Because of the good plasticity of Ag, the Ag NWs showed no significant fracture or crack. A high-magnification image showed that slip bands occurred close to the indent area after deformation, as highlighted by the arrows in Fig. 1e. From the measured angle (60°) between these slip bands and the long axis of the Ag NWs [011], the slip direction was identified as <110>, which is the common slip direction of face-centered cubic (FCC) materials [29, 30]. In the indent (see Fig. 1f), the slip bands were invisible because of the large deformation and confinement of the indenter tip. Interestingly, one side of the Ag NWs showed a “*V*” groove, which might have been due to the restriction of the slip directions during deformation. This was also observed in other indents.

**Fig. 2:**
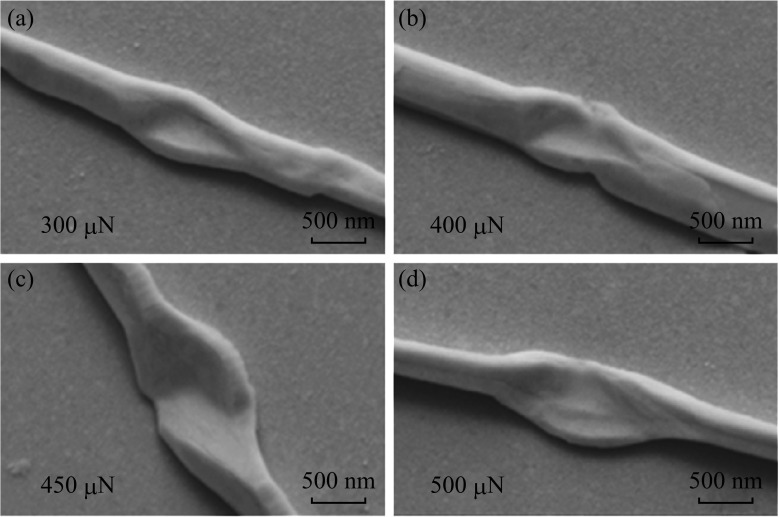
Microstructures of indentation areas after bonding with different forces from 300 to 500 µN

Different bonding morphologies were observed when the force was changed. Figure 2 shows the results with bonding forces of 300–500 µN. When the force is low, the indent is small, and the widening of the thickness is not obvious, as shown in Fig. 2a, b. The deformation is also transmitted away from the indented area. As the force increases to 450 µN or more, the distinct widening indicates a large deformation (see Fig. 2c, d). Since further increasing the force might lead to a displacement of the indenter tip close to or larger than the thickness of the Ag NWs, the plastic deformation could penetrate into the Au electrode, which is not desirable in electronics. Because the thicknesses of the Ag NWs and Au electrode were quite small (all on the nanoscale) and the strain of the Ag NWs was more than 50% in the thickness direction, the measured hardness would be greatly affected by the substrate. Here, we did not calculate the reduced hardness and module using the Oliver-Parr relation [31–33].

**Fig. 3:**
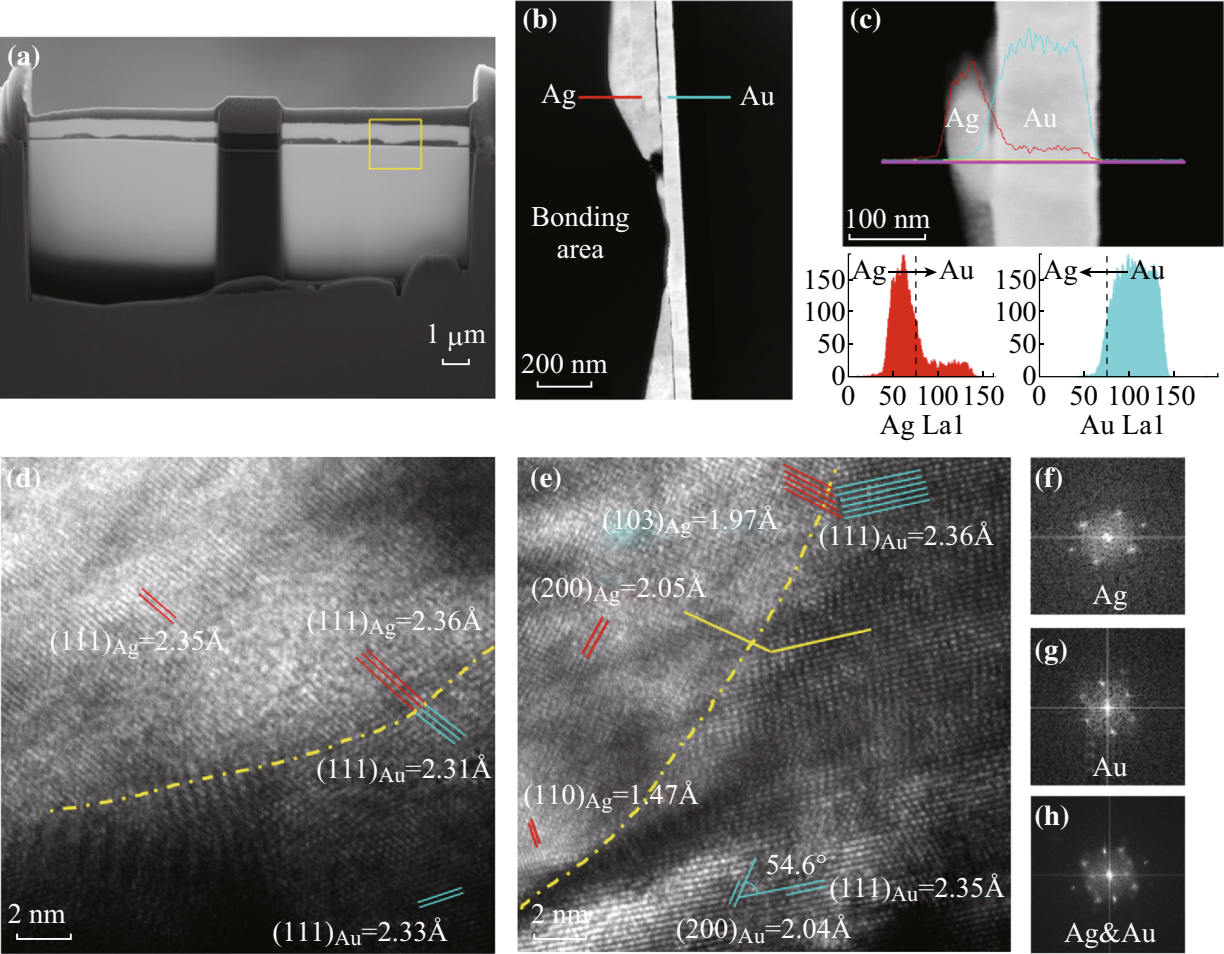
**a** SEM image of FIB sample after thinning. **b** The STEM image of a Ag–Au cross section after wire bonding (with 450 µN) sliced with FIB. **c** The line profiles of the Ag and Au across the Ag–Au interface from the STEM image. **d** HRTEM image of Ag–Au interface with matched (111) lattices showing both the Ag and Au sides. **e** The severely deformed lattices on the Ag and Au, with the Ag showing a 4H lattice (103) at the interface. The FFT images were taken from **e** showing the patterns of **f** the Ag side, **g** Au side, and **h** interface

To observe the Ag–Au interface after bonding, the indent was cut using the FIB (see Fig. 3a). The cross section was less than 100 nm in the TEM observation, as shown in Fig. 3b. The Au only had a slight deformation after bonding using a force of 450 µN. This meant that the substrate could be less affected using this wire-bonding method compared to conventional techniques. Because the thickness of the Ag NWs was quite small and the resolution of the FIB was 20 nm, the cross section was slightly off center. However, it still clearly shows the Ag–Au interface. Figure 3c depicts the EDS line scanning profile of the Ag and Au elements under the scanning transmission electron microscope (STEM) mode. Under a massive localized pressure, the organic protective layer could be broken and allow the Ag atoms to diffuse into the Au at room temperature. Furthermore, the pressure also promoted interdiffusion. It was found that the Ag diffused further into the Au than the Au did into the Ag. This might have been a result of the different diffusion rates for the Ag in Au and the Au in Ag. The Arrhenius equation *D* = *D*_0_ exp(−*Q*/RT) indicates that the diffusion coefficient *D*, frequency factor *D*_0_, and activation energy *Q* determine the diffusion rate when the temperature is constant. Here, the wire-bonding process was completed at room temperature. The activation energy of diffusion for Ag in pure Au is 40.2 kcal (mole)^−1^, whereas that of Au in pure Ag is 48.3 kcal (mole)^−1^; *D*_0_^Ag→Au^ was 0.07 cm^2^ s^−1^, and *D*_0_^Au→Ag^ was 0.85 cm^2^ s^−1^ [34]. At 820 °C, *D* (Ag → Au) was 6.01 × 10^−10^ cm^2^ s^−1^ and *D* (Au → Ag) was 1.88 × 10^−10^ cm^2^ s^−1^ [35]. Thus, it could be speculated that the Ag diffused into the Au more than the Au did into the Ag at the same temperature.

At room temperature, according to the measured diffusion distances shown in Fig. 3c, *d*_Ag→Au_ ≈ 66.1 nm and *d*_Au→Ag_ ≈ 13.9 nm. In one dimension, the diffusion length *d* can be written as *d* = 2(*Dt*)^1/2^ as Fick’s law of diffusion. Consequently, the effective diffusion coefficients $D_{{e{\text{ff}}}}^{{{\text{Ag}} \to {\text{Au}}}}$ (Ag in Au) and $D_{\text{eff}}^{{{\text{Au}} \to {\text{Ag}}}}$ (Au in Ag) could be simply calculated using *D*_eff_ = *d* ^*2*^ */*4*t*, where *d* is the diffusion distance in one dimension measured using Fig. 3c. The diffusion time *t* was difficult to determine because a large pressure was applied to promote diffusion during bonding (the total loading and unloading time was 45 s) and then for six days (May 3rd to 9th) for the TEM observation (roughly 500,000 s in total). Therefore, $D_{\text{eff}}^{{{\text{Ag}} \to {\text{Au}}}}$ and $D_{\text{eff}}^{{{\text{Au}} \to {\text{Ag}}}}$ were 2.43 × 10^−6^ and 1.07 × 10^−7^ cm^2^ s^−1^, respectively, when only considering the 45 s loading–unloading time; and 2.18 × 10^−10^ and 9.66 × 10^−12^ cm^2^ s^−1^, respectively, when considering the total time at room temperature. These values provide a reference showing that the diffusion at the nanoscale would be quite high even at room temperature. On the other hand, the atomic radius of Ag (0.165 nm) is smaller than that of Au (0.174 nm), while their lattice constants are almost the same (0.408 nm for Ag and 0.408 nm for Au), causing the Ag to diffuse into Au more easily. Moreover, because the Au electrodes were sputtered with polycrystalline, the existence of large grain boundaries and vacancies were expected compared with polyol-synthesized Ag NWs. However, because the Ag NWs that were synthesized using the polyol method had a structure that consisted of five single crystalline prisms bonded with five twin boundaries [19], there were far fewer defects. These grain boundaries and defects could be attributed to the larger diffusion rate of the Ag into the Au compared to that of the Au into the Ag. The pressure may have played a very important role in promoting the diffusion, “squeezing” the Ag into the Au more easily than “squeezing” the Au into the Ag.

The HRTEM images in Figs. 3d and e show that the Ag–Au interfaces formed a diffusion-metallurgical bond. No pores are observed at the interface. The moiré fringes in Fig. 3d indicate that dislocations formed after bonding, induced by the large deformation. For some locations, the (111) lattice of the Ag aligned with the (111) of the Au (see Fig. 3d). Moreover, other locations without good lattice matching (perhaps because they were not in the right zone axis) are also identifiable and without voids (see Fig. 3e). At the Au side, the 2.04 and 2.35 Å values show the (200) and (111) lattices of Au, respectively. Interestingly, the (103) lattice at the Ag side shows a 1.97 Å distance, which might belong to the 4H crystalline structure of Ag. Such a 4H structure could be found in Ag NWs [36] and Au nanoribbon [37] when their synthesis conditions were restricted. This metastable phase could transform into the FCC structure through the displacement of Ag atoms using an electron beam [36]. The fast Fourier transform (FFT) images on the Ag and Au sides and their interface are shown in Fig. 3f–h, respectively. These FFT patterns confirm the crystalline structure of the FCC structures after bonding, even if there is a little Ag getting into the 4H structure.

## Conclusion

In summary, Ag NWs could be controllably bonded onto a Au substrate by applying pressure with the assistance of nanoindentation at room temperature. The Ag NWs showed good ductility, and severe slip bands were observed after deformation. Without external heat input, the plastic deformation could break the organic shell on the surface of the Ag nanowires and form an atomic contact at the Ag–Au interface. A metallic bond was formed in this room temperature wire bonding process. The interface displayed no pores, but showed lattice matching on the (111) plane and large lattice mismatching at others. This nanoscale wire-bonding process might present opportunities for future nanodevice integration or nanoscale electronic packaging.
